# Case of Imperfect Rectum, for Which an Operation Was Successfully Performed

**Published:** 1826-09

**Authors:** 


### MALFORMATION OF THE RECTUM.

*Case of Imperfect Rectum, for which an Operation was successfully performed.* By Mr. EARLE.

A CHILD, aged nine days, was brought to St. Bartholomew's Hospital, June 17, 1826, in consequence of an imperfect rectum. The abdomen was so much distended, that the convolutions of the intestines could be distinctly traced through the parietes. Mr. Earle examined the child, and found the anus perfectly formed, and a cul de sac about an inch and a half in length, into which he inserted his little finger. In doing this, he stated that he felt the sphincter muscle contract strongly; and at the same time a strong impulse from within was conveyed to the top of his finger. He passed the canula of a full-sized trochar, and pressing it against the bottom of the sac, he introduced the trochar, and, after piercing about three lines in depth, the canula passed freely into the bowel, and the trochar was withdrawn. A powerful discharge of flatus followed, which was succeeded by well-coloured feculent matter. The canula was retained in for forty-eight hours; after which an elastic gum tube was substituted for some days, and the passage was directed to be enlarged by the introduction of bougies. The child recovered perfectly.

The point of most interest, next to the successful termination after the lapse of nine days, was the total absence of meconium, of which not a vestige could be traced in the fæces, which were remarkably healthy in colour and consistence.

